# Supplementary material for: Surface Motility Regulation of Sinorhizobium fredii HH103 by Plant Flavonoids and the NodD1, TtsI, NolR, and MucR1 Symbiotic Bacterial Regulators
Source: Int J Mol Sci. 2022 Jul 12;23(14):7698. doi: 10.3390/ijms23147698 (PMC9316994; doi:10.3390/ijms23147698)
Supplement: Supplementary file 1 [file ijms-23-07698-s001.zip › ijms-1808061-supplementary.pdf]

Surface Motility Regulation of *Sinorhizobium fredii* HH103 by Plant Flavonoids and the NodD1, TtsI, NolR, and MucR1 Symbiotic Bacterial Regulators

Cynthia Alías-Villegas[1], Francisco Fuentes-Romero[2], Virginia Cuéllar[3], Pilar Navarro-Gómez[2], María J. Soto[3], José-María Vinardell[2,*], Sebastián Acosta-Jurado[1,*]

List of Supplementary Material

Figure S1. Growth curves of *S. fredii* HH103 Rif^R and different mutant derivatives in liquid MM in the presence (**A**) or absence (**B**) of genistein.

Figure S2. Analysis of genistein-induced surface motility on *S. fredii* HH103 additional mutants in the *nodD1, ttsI, nolR* and *mucR1* genes.

Table S1. *S. fredii* HH103 derivatives used in this study.

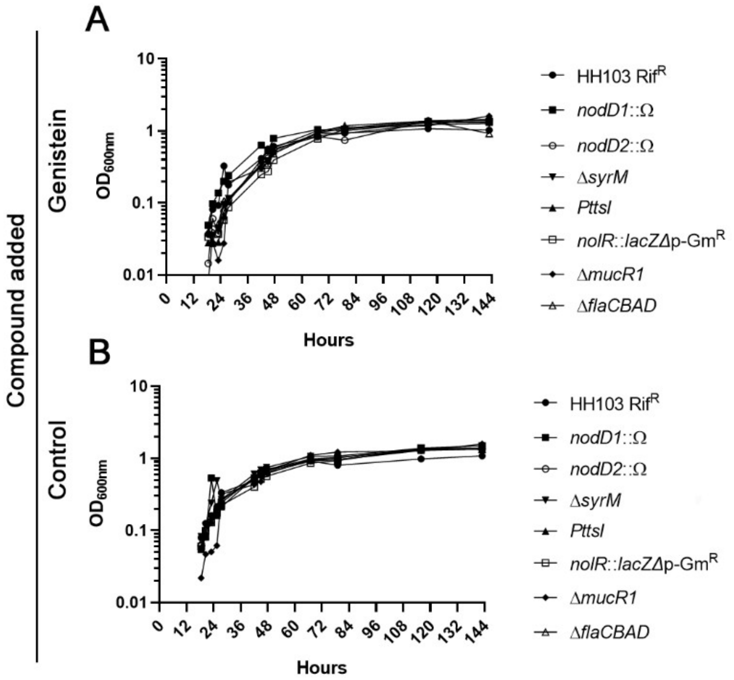

Figure S1. Growth curves of *S. fredii* HH103 Rif^R and different mutant derivatives in liquid MM in the presence (**A**) or absence (**B**) of genistein.

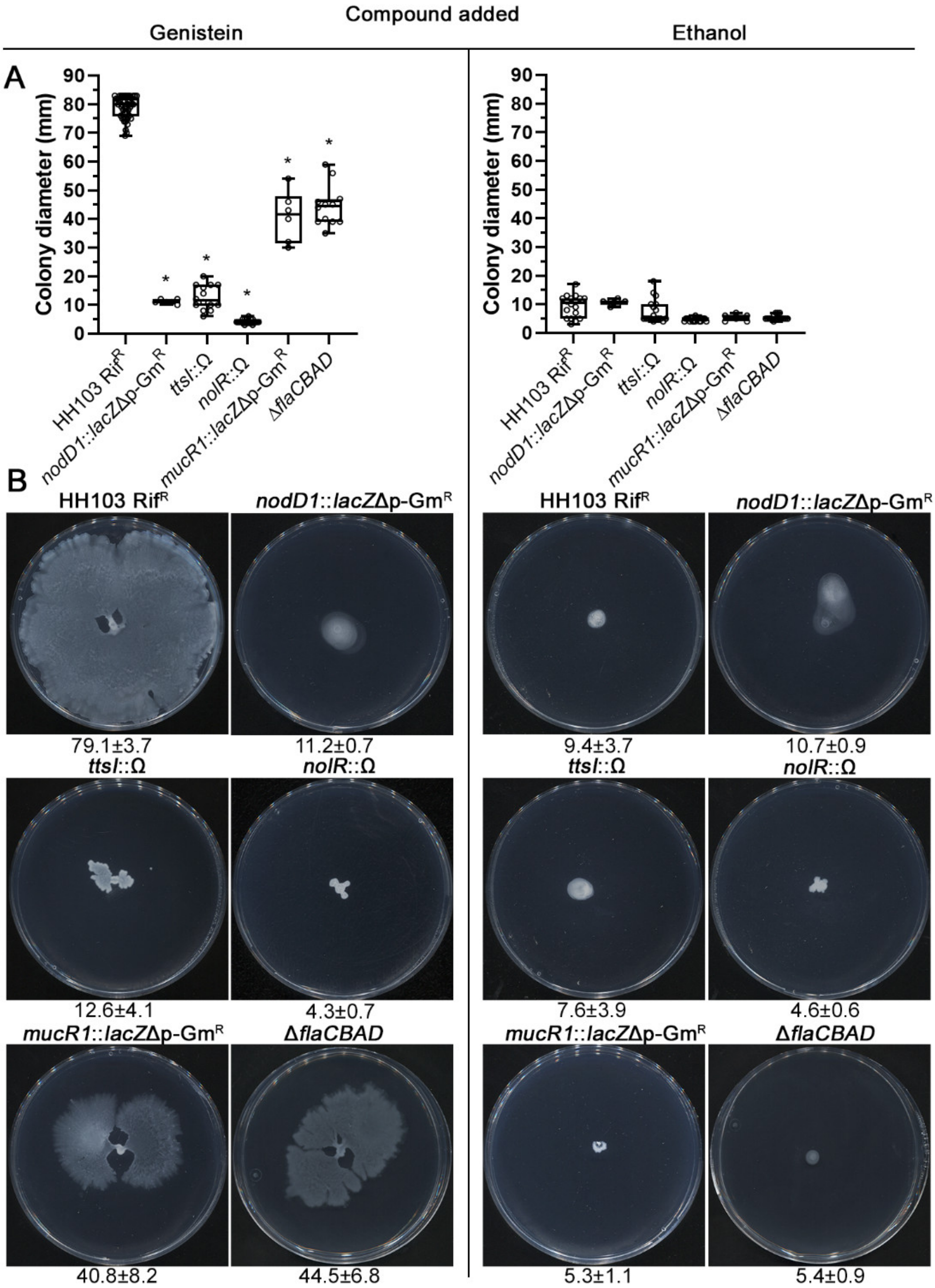

Figure S2. Analysis of genistein-induced surface motility on *S. fredii* HH103 additional mutants in the *nodD1*, *ttsI*, *nolR* and *mucR1* genes. The non-flagellated (*flaCBAD*) mutant was also included. Surface motility experiments were performed on minimal medium (MM) using 0.4% agarose as gelling agent at 72 hours. **A.** Box and whisker plots from at least three biological replicates performed with three technical replicates. Asterisks indicate significant differences with the corresponding control sample using the non-parametric test of Mann-Withney, α = 1%. **B.** Representative pictures of the motilities exhibited in the presence of genistein by the different *S. fredii* HH103 mutants analysed. Values under images represent the average and standard deviation of surface migration (given in millimetres and determined as described in the text).

Table S1. *S. fredii* HH103 derivatives used in this study.

Derivative	Source or reference
HH103 Rif^R Δ*flaCBAD*	[52]
HH103 Rif^R Δ*gunA*	[62]
HH103 Rif^R Δ*mucR1*	[19]
HH103 Rif^R *mucR1*:: *lacZΔp-Gm^R*	[19]
HH103 Rif^R *nodA*::Tn5-*lacZ*	[54]
HH103 Rif^R *nodD1*:: *lacZΔp-Gm^R*	[55]
HH103 Rif^R *nodD1*::Ω	[53]
HH103 Rif^R *nodD2*::Ω	[20]
HH103 Rif^R *nolR::lacZΔp-Gm^R*	[24]
HH103 Rif^R *nolR*::Ω	[17]
HH103 Rif^R *nopA::lacZΔp*	[17]
HH103 Rif^R *nopC::lacZΔp*	[61]
HH103 Rif^R *nopD*::Ω	[63]
HH103 Rif^R *nopI*::Ω	[58]
HH103 Rif^R *nopL*::Ω	[58]
HH103 Rif^R *ΔnopM1-nopM2:: lacZΔp-Gm^R*	[63]
HH103 Rif^R *nopP*:: *lacZΔp-Gm^R*	[59]
HH103 Rif^R *nopT*::Ω	[63]
HH103 Rif^R *rhcJ*:: Ω	[56]
HH103 Str^R *rhcJ*::Tn5-*lacZ*	[56]
HH103 Str^R *rhcV*:: Ω	[57]
HH103 Rif^R Δ*syrM*	[21]
HH103 Rif^R p*ttsI*::Ω	[17]
HH103 Rif^R *ttsI*::Ω	[17]
